# A Breeding Plumage in the Making: The Unique Process of Plumage Coloration in the Crested Ibis in Terms of Chemical Composition and Sex Hormones

**DOI:** 10.3390/ani13243820

**Published:** 2023-12-11

**Authors:** Danni Liu, Yiwei Tong, Rong Dong, Xinping Ye, Xiaoping Yu

**Affiliations:** 1 College of Life Sciences, Shaanxi Normal University, Xi’an 710119, China; danny19@yeah.net (D.L.); tongyiwei2023@163.com (Y.T.); yexinping@snnu.edu.cn (X.Y.); 2 Research Center for Qinling Giant Panda, Shaanxi Academy of Foresty, Xi’an 710082, China; donglittle@sohu.com; 3 Research Center for UAV Remote Sensing, Shaanxi Normal University, Xi’an 710119, China; 4 Shaanxi Provincial Field Observation and Research Station for Golden Monkey, Giant Panda and Biodiversity, Xi’an 723400, China

**Keywords:** Crested Ibis, daubing behavior, black substance, plumage color change, sex hormones

## Abstract

**Simple Summary:**

The Crested Ibis (*Nipponia nippon*) exhibits a unique behavior to develop its nuptial plumage coloration. In our study, we have discovered a significant correlation between sex hormone levels, the secretion, as well as the daubing behavior of this black substance. Furthermore, we identify multiple chemical compounds present in the black substance and to analyze their contribution to its plumage coloration. These findings provide a solid foundation for the further exploration and understanding of the biological significance of the black substance.

**Abstract:**

The Crested Ibis (*Nipponia nippon*) has long fascinated ornithologists with its enigmatic plumage color change. After more than a century of curiosity, the mystery was finally unraveled in the 1970s, unveiling the mechanism behind this remarkable transformation. Unlike other bird species, the Crested Ibis achieves its nuptial plumage coloration through a unique daubing behavior. After a water-bathing, it applies a sticky black substance secreted by a patch of skin in the neck and throat region. However, the chemical components of this black substance have not been studied in detail until now. To address this issue, we conducted a study to detect the components of the black substance and explore the relationship between sex hormone levels and the secretion of the black substance. We used enzyme-linked immunosorbent assay (ELISA) to measure the monthly changes in steroid hormone levels (estradiol E2, testosterone T, and progesterone PROG) levels in feces. We also analyzed the correlation between sex hormone levels and daubing behavior. The results showed that the sex hormone levels are closely related to the secretion and application of the black substance. In addition, we qualitatively analyzed the chemical components of the black substance using gas chromatography–mass spectrometry (GC-MS), uncovering the presence of 117 distinct chemical components. We assume that the black coloration results from the polymerization of selected chemical constituents among these components. These findings provide a groundwork for further exploration into the biological significance of the black substance. Overall, our study detected components in the black substance and studied how sex hormone levels relate to its secretion. Understanding the hormone effects on coloration helps in precise habitat management, like wetland preservation, crucial for Crested Ibis survival. Implementing hormone-boosting measures during breeding seasons enhances reproduction and health, vital for their conservation.

## 1. Introduction

The Crested Ibis (*Nipponia nippon*) is an endangered bird species native to East Asia, including China, Korea, Japan, and Russia. Unfortunately, habitat loss, hunting, and excessive pesticide use have driven a rapid decline in the global population of Crested Ibises. By the mid-20th century, they had vanished from the wild in the former Soviet Union, the Korean Peninsula, and Japan [1]. However, the discovery of a small population of seven wild Crested Ibises in Yaojiagou, Yang County, Shaanxi Province, in 1981, marked the sole surviving wild population globally [2]. This led to its classification as a critically endangered species and a National Class I key protected animal. Over the past three decades, conservation efforts have yielded significant results. The endangered status has shifted from critically endangered to endangered, with the current Crested Ibis population reaching 11,000 individuals. Scientists are actively conducting research in various aspects including population dynamics [3,4], genetics [5,6,7], disease management [8,9], habitat restoration [10,11,12], conservation efforts [13,14], and behavior observation [15]. One particularly intriguing aspect of this species is its distinct plumage color changes that occur throughout the year, especially during the breeding season. Understanding the factors that drive these plumage transformations is crucial for gaining insights into breeding behavior, health assessments, and developing effective conservation strategies [16].

Birds have evolved a wide range of colors and patterns in their feathers, making them visually captivating creatures. Bird coloration, including vibrant plumage, believed to reflect individual quality, is essential for various aspects of avian life, such as protection, camouflage, mate selection, signal recognition, survival, reproduction, and species recognition [17,18,19,20]. Ornithologists have long been fascinated by the mechanisms behind these colors and patterns as they provide valuable insights into the heredity, variation, and evolution of bird species. As research on bird plumage coloration has progressed, it has become increasingly specialized. From its initial focus on visual perception, species recognition, sexual selection, and biological metabolism [21], the study of bird plumage coloration has expanded to encompass a wide range of disciplines, including ecology, physiology, genetics, and evolution [22,23]. Bird coloration can be broadly classified into two main types based on their formation mechanism: pigment-based coloration and structural coloration. Pigment-based coloration in birds is dependent on pigments such as carotenoids and melanin [24]. Carotenoids, which are responsible for producing vibrant red, orange, and yellow hues, are obtained from the environment, primarily through the bird’s diet. Unlike some other animals, birds cannot synthesize carotenoids on their own [25,26,27]. While melanin is indeed synthesized by birds themselves, it is important to note that structural coloration is a distinct mechanism for producing colors in bird plumage. Structural coloration relies on the microstructure of feathers to control the refraction and interference of light, resulting in a wide array of colors known as iridescence [28].

**Figure 1:**
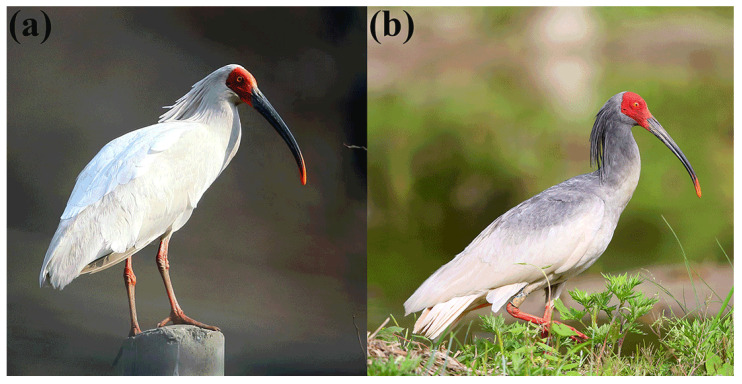
Variation in the plumage color of Crested Ibis in (**a**) non-breeding and (**b**) breeding season. The plumage color is white in the non-breeding season while changing to gray in the breeding season.

The coloration mechanism of the Crested Ibis is classified as melanin-based coloration [29]. This bird species exhibits distinctive seasonal variation in plumage coloration between breeding and non-breeding seasons. However, over a century ago, ornithologists were unaware of the mechanism behind the Crested Ibis’s plumage coloration, leading to a long-standing controversy about the existence of the white and gray pattern [30]. It was not until the 18th century, when Berezovskii discovered that the gray feathers were actually a change in plumage color during the breeding season of the Crested Ibis [31]. The dispute was ultimately resolved in the 1970s [32], confirming that the Crested Ibis achieves plumage color change through a daubing behavior. This daubing behavior plays a significant role in a unique form of cosmetic adaptation classified as a skin secretion type [33]. This unique cosmetic coloration process involves the repeated application of a black powdery substance, secreted from the neck skin, onto the head, neck, back, and shoulders following a water bath [32]. This process results in the formation of the birds’ nuptial plumage (Figure 1). In the field of avian plumage research, a strong connection has been established between plumage coloration in various bird species and sex hormones. For instance, testosterone plays a role in influencing the production of carotenoid pigments [34] and melanin [35]. Therefore, exploring the interplay between melanin and sex hormones is pivotal for uncovering the intricate mechanisms driving the plumage transformations observed in Crested Ibises. However, to date, limited investigation has been conducted to explore the association between the secretion of the black substance and sex hormone levels in the Crested Ibis. The black substance is composed of a liposoluble matrix and contains melanin [32]. Nonetheless, the precise chemical composition of this black substance has yet to be determined. Therefore, the main research objective of this study is to examine the chemical composition of the black substance and investigate its relationship with sex hormone levels. By doing so, our study aims to provide a comprehensive understanding of the intricate mechanisms underlying the Crested Ibis’s unique coloration process and explore its potential ecological significance.

## 2. Materials and Methods

### 2.1. Study Samples

To assess monthly changes in sex hormone levels, a group of seven adult Crested Ibises, including three males and four females, were obtained from the Shaanxi Rare Wildlife Rescue and Breeding Center. Each enclosure for captive Crested Ibises accommodates around 3–4 birds, arranged in a row of cages. To minimize disruption and impact on other ibises, we specifically opted for the two outermost cages in the row. The age and gender of each ibis were determined by observing the color plastic rings and metal numbered rings on their legs, allowing for rapid and accurate identification. Sampling was conducted in enclosures located in close proximity to minimize interference with other ibis individuals. Due to these constraints, specific selections based on age and gender were not possible. However, efforts were made to ensure a diverse representation within the group. The relevant data for each individual, including their color plastic ring numbers (CPRN), gender (3 males and 4 females), birth year, metal ring numbers (MRNs) and feeding areas were presented in Table 1. Each individual was uniquely identified by a specific color plastic ring number: 543, 491, 461, 443, 334, 603, and 527, respectively. The average age of the entire group was 12.1 ± 1.35 (*n* = 7) years, with both males and females ranging in age from 10 to 14 years.

### 2.2. Cosmetic Behavior of Crested Ibis

The Crested Ibis exhibits crucial ecological behaviors, involving water bathing and daubing, which have significant implications for species’ survival and conservation. Daubing behavior, which is believed to be a form of communication, may play a role in social bonding and territorial marking [36]. Our field observations have provided insights into the patterns of these behaviors. Based on our observations, we have noted that the Crested Ibis water-bathing behavior typically occurs during the crepuscular periods of the day, specifically in the early morning and late afternoon. On the other hand, the daubing behavior is more commonly observed during periods of strong sunlight in daytime. To observe these behaviors, we conducted long-distance observations using binoculars (SICONG 10 * 42 mm) during two specific daily periods. The morning observation period was from 9:00 a.m. to 11:00 a.m., while the afternoon observation was from 2:00 p.m. to 5:00 p.m. These time frames were chosen to capture the peak activity of water-bathing events and daubing behaviors.

### 2.3. Collection of Feces and Black Substance

In this study, we utilized a non-invasive trace sampling method to collect fresh feces from seven Crested Ibises for the detection of three steroid hormones: estradiol, testosterone, and progesterone [37]. The behavior monitoring and fecal sample collection took place from January to December 2017. To ensure consistency and minimize disturbances, fecal samples were collected in the middle of each month at a specific time frame after the morning feeding, precisely between 9:00 a.m. and 10:00 a.m. This timing was chosen intentionally, considering the regular feeding schedule of the Crested Ibises, which facilitated the sampling process. To prevent soil contamination during collection, we placed a transparent plastic sheet directly underneath the wooden perches where the Crested Ibises typically rested. This strategic placement helped in collecting the fecal samples promptly and maintaining their integrity. Each fecal sample was retrieved immediately upon excretion and stored in a −20 °C low-temperature refrigerator throughout the experiment. The refrigerator model used was BCD-215YD E (Qingdao Haier Co., Ltd., Qingdao, China). On average, the samples were stored for approximately one week before further analysis. Detailed information regarding each fecal sample, including the individual identification number (CPRN), date of collection, temperature, and fecal sample type, was recorded for accurate tracking and analysis purposes.

**Figure 2:**
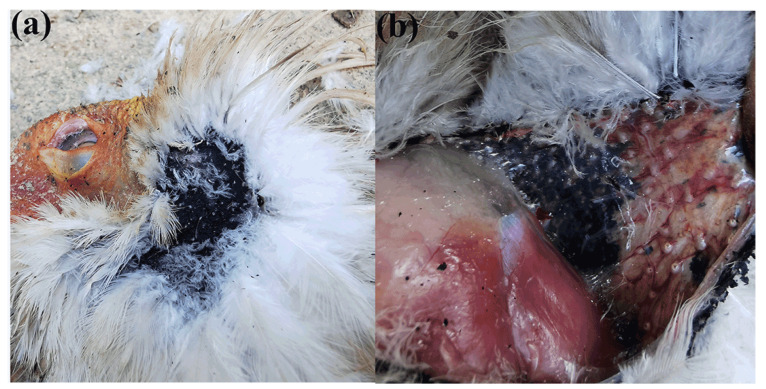
Black substance secreted by the neck skin (black) of the Crested Ibis. (**a**) The black skin on the neck of the Crested Ibis; (**b**) The black skin containing intracellular black granular substance.

To examine the chemical constituents of the black substance, we expanded our sample collection to include two additional Crested Ibises (CPRN: 307; 501) during the breeding season. The reason we did not use the same seven Crested Ibises is that they are exceptionally sensitive to external disturbances during the breeding season. We collected samples from the necks of these two Ibises and incorporated the relevant parameters into Table 1 to include their information. In Figure 2, we depicted the location where the black substance is secreted in the Crested Ibis, along with our collection process. Figure 2a shows the black skin area located on the neck, while Figure 2b provides visual evidence of the presence of intracellular black granular substances within this black skin area. To ensure a comprehensive analysis, we also collected samples from frozen corpses in breeding plumage. By including samples from both live individuals and frozen corpses, we can analyze the chemical composition of the black substance across the different stages of the breeding season. This approach helps ensure that our results are representative of the whole species and provide a more complete understanding of the chemical constituents of the black substance.

### 2.4. Qualitative Analysis of the Sex Hormone Level and the Black Substance

To extract the hormones from the fecal samples, we used an equal amount (0.5 g) of standard solution. The levels of the steroid hormones in Crested Ibis feces were measured using enzyme-linked immunosorbent assay (ELISA). For this study, we employed 96-well ELISA kits (Shanghai Xi tang Biotechnology Co., Ltd., Shanghai, China) with a measurement range of 0–1 ng/mL for estradiol, 0–20 ng/mL for testosterone, and 0–50 ng/mL for progesterone, respectively.

To detect the chemical composition of samples, we used gas chromatography–mass spectrometer (GC-MS), which involved two steps. Firstly, we conducted an extraction of volatile and semi-volatile components from the samples. This process was carried out by dissolving the samples and subjecting them to various techniques such as ultrasonic extraction, vortex oscillation, centrifugation, dilution, and filtration. Secondly, we performed the ultrasonic extraction and analysis of the samples. Prior to the examination, we took 0.01 g samples of the black substance and placed them in a 1.5 mL EP tube. To mitigate the adverse effects of high temperature produced by ultrasound, we used a beaker filled with dry ice during the ultrasonic analysis, which was conducted for a duration of 30 min, employing a power of 120 W.

### 2.5. Statistic Analysis

We employed OriginPro 95E software (OriginLab Corporation, Northampton, MA, USA) for our statistical analysis. The standard deviation (SD) of the sample is calculated using the formula: $SD=\sqrt{\frac{\sum_{i=1}^{n}{\left(x_{i}-\mu \right)}^{2}}{n}}$, where $x_{i}$ represents the value of each sample, μ is the average sample value, and *n* denotes the sample size. The correlation coefficient (r), assessing the linear relationship between two continuous variables, is derived from the formula: $r=\frac{\sum \left(h-\bar{h}\right)\left(d-\bar{d}\right)}{\sqrt{\sum {\left(h-\bar{h}\right)}^{2}\;\cdot \;\sum {\left(d-\bar{d}\right)}^{2}}}$, where *h* and *d* represent the observed values of the sex hormone level and daubing frequency, respectively. $\bar{x}$ and $\bar{d}$ indicate the means of the respective variables. The *p*-value was computed using the t-test method. The T-value is typically calculated as $t=\frac{r\;\cdot \;\sqrt{n-2}}{\sqrt{1-r^{2}}}$. Once the t-value is obtained, the *p*-value can be calculated using the degrees of freedom and a t-distribution table, where the degrees of freedom are generally df=n−2.

## 3. Results

### 3.1. Cosmetic Behavior and Sex Hormone Levels

From late January through April, just prior to the breeding season, we observed a specific behavior in the Crested Ibis and conducted a statistical analysis. The sample size for this analysis was n=7, with degrees of freedom df=4. After water bathing, the ibises exhibited a characteristic behavior of rubbing the side of their head to the neck, back and shoulder region with noticeable frequency. This behavior, on average, lasted for 6.5 ± 2.8 (n=7) min and was followed by regular preening.

**Figure 3:**
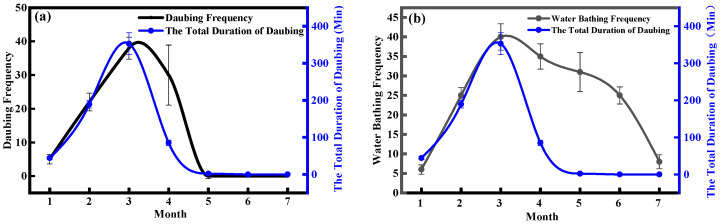
(**a**) Monthly variation in daubing frequency and total daubing duration. (**b**) Monthly variation in bathing frequency and total daubing duration. The frequency of bathing, daubing, and total duration time exhibit a consistent increase starting from January, reaching their peak in March, and subsequently gradually decreasing.

During our study, we recorded the frequency of water bathing and daubing, as well as the duration of daubing behavior in the Crested Ibis. The data pertaining to these observations are presented in Table 2. To provide a clearer representation of the changes in daubing frequency and the total daubing duration from January to July, we plotted the corresponding curves in Figure 3a. The results show a consistent upward trend in daubing behavior, both in terms of frequency and total duration, from January to March. The behavior reached its peak in March and subsequently exhibited a gradual decline from March to May.

**Figure 4:**
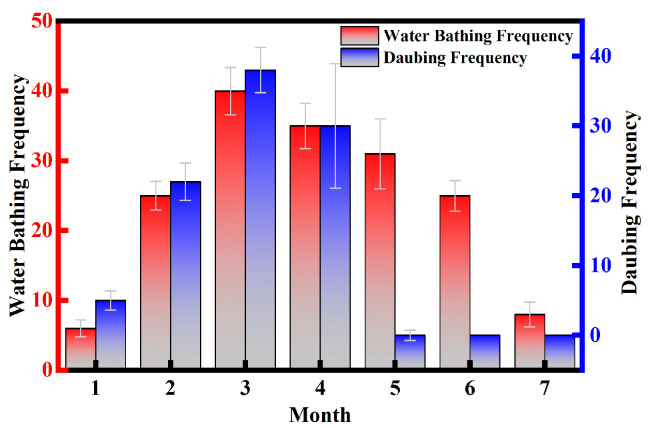
Water bathing and the daubing frequency of Crested Ibis. Both water bathing and daubing behaviors peak in March. During the months of May–July, daubing behavior was absent, while the water bathing behavior remained consistent.

The water bathing frequency and daubing frequency of the Crested Ibis reached their maximum in March. From January to March, there was a positive correlation (r=0.56,p=0.196) between the duration of daubing behavior in Crested Ibis and the corresponding increase in water bathing frequency (Figure 3b). Notably, from May to July, the daubing behavior was absent, while the water bathing behavior remained consistent (Figure 4). We also observed that the coloring of the Crested Ibis was at its deepest shade in March, which positively correlated with the frequency of water bathing and daubing behavior (r=0.67,p=0.17). These findings suggest that the color changes mainly occur during February and March. It is evident from these data that there is a clear relationship between the frequency of daubing behavior and plumage color change. Specifically, the frequency and duration of the daubing behavior may influence the amount of black substance applied to the feathers, thereby affecting the intensity of the cosmetic coloration.

**Figure 5:**
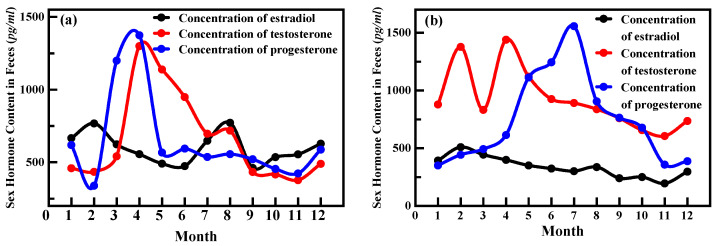
Monthly changes of sex hormone levels in (**a**) female and (**b**) male. For females, estradiol, testosterone, and progesterone secretion peak from January to May. In males, estradiol and testosterone levels rise during from January to May, while progesterone peaks in July.

Figure 5 illustrated the variations in sex hormone levels of Crested Ibis from January to July. The monthly assessments of sex hormone levels reveal a pattern in the estradiol levels of female Crested Ibis. These levels gradually increase starting from the early stages of courtship in December, reaching their peak in February during the courtship period (Figure 5a). Subsequently, the estradiol levels gradually decrease until the end of the reproductive period, with a high increase observed in August. Throughout the roaming period (July, August, September, and October), the estradiol content of the Crested Ibis remains stable. Contrasting the estradiol pattern, the testosterone secretion in female Crested Ibis is low during the initial stages of courtship and mating periods (January, February, and March). However, from April until the end of the breeding period, the testosterone secretion levels are relatively high, with a major peak occurring in April. Following the breeding season, the testosterone content of female Crested Ibis also decreases correspondingly. In general, the secretion of progesterone in female Crested Ibis becomes active after the mating period. Specifically, the secretion of progesterone exhibits a continuous increase from the end of the courtship period until April, when the hatching period begins (March and April). During this period, the secretion of progesterone reaches its peak and subsequently gradually decreases. However, there is a slight decrease in progesterone secretion prior to the end of the reproductive period, followed by a significant decline after the reproductive period. Moreover, the total secretion of estradiol in female Crested Ibis is relatively low, while testosterone secretion is high during the courtship period, with a small peak observed in February.

The sex hormone dynamics in male Crested Ibis (Figure 5b) exhibit similarities to those in females. Estradiol secretion follows a comparable pattern, with levels gradually increasing from the early stages of courtship, peaking in February, and gradually decreasing thereafter. Testosterone secretion in male Crested Ibis is particularly elevated in February and April, with a great decrease in March, followed by a gradual decline after April. Throughout the non-reproductive period, male Crested Ibises maintain consistently low hormone levels. In terms of progesterone levels, they are initially low during the courtship period but gradually increase throughout the reproductive period, reaching their peak in July.

### 3.2. Chemical Components of the Black Substance

**Figure 6:**
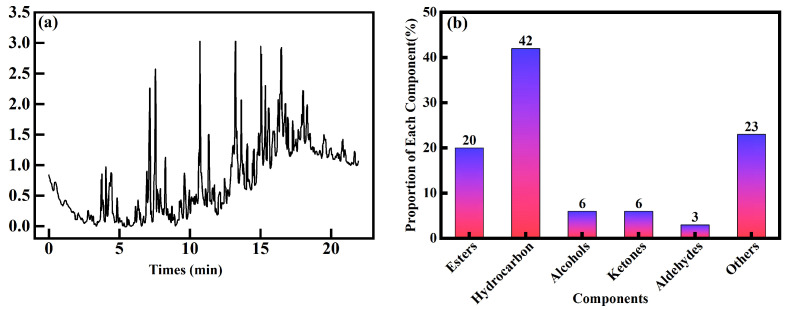
(**a**) Mass spectrometry of chemical composition of the black substance. Mass spectrometry reveals black substance composed of 117 chemical compounds. (**b**) The proportion of chemical component in the black substance. Black substance including 23 esters, 50 hydrocarbons, 7 alcohols, 7 ketones, 3 aldehydes, and 27 other components, accounting for 20%, 42%, 6%, 6%, 3%, and 23%. These proportions represent the percentage of each substance category in the total amount of the black substance.

Figure 6 shows the chemical composition of the black substance in Crested Ibis. In (Figure 6a), we present the mass spectrometry analysis of the black substance, which showcases its chemical makeup. The black substance, which is typically observed in a powdery or flaky form, serves partially as a fat-soluble emulsifier. To gain a deeper understanding of its composition, we conducted a quantitative analysis of the main components present in the black substance. Our results showed that this secretion is a water-insoluble mixture consisting of 117 chemical compounds. These compounds belong to different categories: 23 esters, 50 hydrocarbons, 7 alcohols, 7 ketones, 3 aldehydes, and 27 other components. The relative distribution of these components within the black substance is as follows: esters account for 20%, hydrocarbons make up 42%, alcohols contribute to 6%, ketones comprise 6%, aldehydes contribute to 3%, and the remaining 27 components constitute 23% of the total composition (Figure 6b). Further details regarding the ketones, aldehydes, esters, alcohols, and other components can be found in Table 3. This table provides a comprehensive breakdown of these constituents, including their names and molecular formula.

Esters contribute to the formation of an oil-soluble layer on the feather surface, providing waterproofing and protective attributes. Diverse hydrocarbon compounds among hydrocarbons may influence the distinctive coloration of feathers owing to their absorption characteristics. Alcohols, ketones, and aldehydes fulfill functions in stabilizing the feather color or altering surface properties. Despite the absence of detailed categorization, other components potentially exert a significant influence on the color adjustment and feather property enhancements. The intricate interaction among these diverse compounds likely results in a multifaceted coating on the feathers, potentially offering specific coloration, water resistance, and protective characteristics. This intricate amalgamation aids the Crested Ibis in its adaptation and thriving within its environmental circumstances.

## 4. Discussion

Plumage coloration in birds plays a vital role in various aspects of their biology, including mate selection, camouflage, and territory defense [17,33]. Below, we discussed the mechanism of plumage coloration in the Crested Ibis, with a particular focus on the impact of sex hormones and chemical components. Our results reveal the intricate interplay between hormonal regulation and chemical composition, ultimately contributing to the vibrant colors of these birds.

### 4.1. Sex Hormone and Plumage Coloration

Plumage color change in birds is a complex process influenced by a range of physiological mechanisms, including hormonal regulation [38], genetics [39], and environmental conditions [40]. This study primarily focuses on how hormonal factors substantially influence plumage coloration in the Crested Ibis.

Hormone regulation plays a crucial role in the mechanism of plumage color change in the Crested Ibis. Estrogen and testosterone are two key hormones involved in the development and expression of plumage coloration in birds. Researchers experimented with testosterone levels in both captive and wild male house sparrows to ascertain its influence on male plumage coloration. They observed that heightened testosterone levels delayed molting, leading to a decrease in red coloration and a duller appearance in the feathers of captive birds, despite their intake of carotenoid-rich diets [41]. Further investigations into Charadriiformes and Corvida species showed a direct relationship between testosterone levels and both plumage melanin deposition and melanin dichromatism [35].

As shown in Figure 5, the testosterone secretion of in male Crested Ibises starts relatively early, peaking from February to April. Female Crested Ibis exhibits low testosterone levels during the initial courtship and mating months. However, from March through the breeding season, their testosterone levels rise, reaching a peak in April, and subsequently decrease post-breeding. Previous studies have shown that testosterone can enhance the expression of genes involved in pigment production, resulting in more vibrant and complex plumage coloration [42,43]. The surge of testosterone during the breeding season promotes ornamental plumage change in the Crested Ibis [44]. Comparison with Figure 4 reveals a clear correlation between daubing behavior and elevated levels of sex hormones. The Daubing frequency reaches its maximum in March, coinciding with the relatively high secretion of testosterone in male ibis individuals in February and April. Although there is a slight decrease in testosterone secretion in March, it is likely due to the mating and egg-laying period when male birds may allocate their energy and resources to other tasks, such as assisting in incubation, chick-rearing, and territory defense. However, the testosterone secretion remains higher than usual during this period. For female Crested Ibis, progesterone plays a more critical role during the egg-laying period as it aids in the process of egg production. Therefore, in March, progesterone secretion reaches its peak. After the egg-laying period, there is a rapid increase in testosterone secretion. To establish a clearer relationship between testosterone levels and daubing behavior, we conducted a correlation analysis, yielding a *p*-value of 0.006, indicating a highly statistically significant correlation between testosterone levels and daubing behavior.

The secretion of estradiol in Crested Ibis is also relatively high around February, facilitating successful mating and egg production. Additionally, in August, both estradiol secretion in females and progesterone secretion in males reach their peaks, presumably for the protection of female Crested Ibises and their offspring. The correlation analysis between the estradiol and daubing behavior yielded a *p*-value of 0.039, indicating a significant statistical relationship between them.

In summary, the secretion of testosterone is positively associated with the daubing behavior in the Crested Ibis. These findings suggest a crucial role for sex hormones in driving the distinctive cosmetic coloration mechanism of the Crested Ibis, as this behavior is most pronounced during the breeding season when sex hormone levels are elevated.

In conclusion, the physiological mechanism underlying the plumage color change in birds is complex, involving hormonal regulation and genetic factors. Future research is needed to comprehensively elucidate the intricate molecular mechanisms underlying the regulation of plumage coloration in birds. Such research will have significant implications for the conservation and management of avian species populations.

### 4.2. Chemical Components and Plumage Coloration

Birds can alter their plumage color through six distinct strategies, including feather abrasion [45,46,47,48], microbial activity [49,50], ectoparasites [51], ultraviolet light [52], the accumulation of dirt particles [53], and the application of cosmetics [33,54]. These six strategies can be classified into two primary mechanisms: structural coloration [55] and pigment-based coloration [27]. Structural colors are created by the interaction of light with specific nanostructures. This produces a unique and often iridescent coloration that is less influenced by environmental factors and more strongly under genetic control [27,55,56]. The most common pigment-based colors are produced by carotenoids and melanins. Carotenoid-based colors are assumed to be highly condition-dependent, while melanin-based colors are mainly under genetic control [56]. Carotenoid-based pigments, which are responsible for red and yellow coloration in many bird species, are obtained through the diet and can also be an indicator of individuals’ ability to obtain high-quality food. Melanin-based pigments, which are responsible for black, brown, and gray coloration, are thought to be associated with the immune system and oxidative stress [27]. The Crested Ibis is unique in that it changes its plumage color through the application of black substance to form nuptial plumage [33]. According to this plumage coloration characteristics, it falls into the category of pigment-based coloration. Specifically, its plumage color changes are based on variations in melanin pigmentation.

Black substance is composed of 117 chemical components, mostly ketones, aldehydes, esters, and alcohols made up of carbon, oxygen, and hydrogen atoms. The components of these 117 substances are very similar to the components of eumelanin, a type of melanin. The polymers and aromatic hydrocarbon compounds present in the molecules of eumelanin components effectively absorb light, especially ultraviolet and visible light. This process results in the black coloration of eumelanin [29]. The high polymers of eumelanin are often composed of unit molecules like 5,6-Dihydroxyindole ($C_{8}H_{8}O_{2}$), 5,6-Dihydroxyindole-2-carboxylic acid ($C_{9}H_{9}NO_{4}$), and 5,6-Dihydroxyindole-2-carboxylic acid ($C_{9}H_{9}NO_{3}$) [57]. From Table 3, we can see that the black substance in the Crested Ibis does not contain these substances, but it does contain many similar organic molecules. For instance, certain molecular components resemble ketones and aldehydes, exhibiting similarities to 5,6-Dihydroxyindole. Meanwhile, some molecules, such as (R)-2,4-Dihydroxy-N-(3-hydroxypropyl)-3,3-dimethylbutyramide ($C_{9}H_{19}NO_{4}$), (1S,2S)-(±)-1,2-Diaminocyclohexane ($C_{6}H_{14}N_{2}$), 1, 3,5-Triazine-2,4(1H,3H)-dione, 6-(ethylamino) ($C_{5}H_{8}N_{4}O_{2}$), and 8,19-Secoyohimban-19-oic acid, 16,17,20,21-tetradehydro-16-(hydroxymethyl)-, methyl ester, (15.beta.,16E)- ($C_{21}H_{24}N_{2}O_{3}$), are similar to 5,6-Dihydroxyindole-2-carboxylic acid and 5,6-Dihydroxyindole-2-carboxylic acid. We hypothesize that the organic compounds within these 117 substances can also polymerize similarly to eumelanin, resulting in high polymers that strongly absorb visible light, leading to the black coloration.

### 4.3. Biological Significance of Avian Plumage Coloration

Plumage coloration is a crucial aspect in avian visual communication, serving various functions such as crypsis, competition, and advertisement [27]. Additionally, plumage coloration can serve as an indicator of environmental conditions, individual health, and genetical quality [58]. In the case of the Crested Ibis, alterations in plumage color offer numerous benefits. The main purpose of plumage coloration is mate attraction. Sexual selection allows for choosing the highest genetic quality mate which is reflected in plumage coloration brightness and intensity. The gray plumage of the Crested Ibis is related to the maturity and reproductive period of individuals, serving as their nuptial plumage. The secondary function of the Crested Ibis’s plumage coloration is protective in nature. During the summer, the gray plumage blends with the black bottom mud commonly found in rice fields, providing camouflage and enhancing the Ibises’s survival during egg hatching. Additionally, the white plumage acquired through molting blends with the snowy environments, further augmenting its protective function. As a result, the changes in plumage coloration in Crested Ibis hold a dual biological significance: nuptial plumage and protective coloration.

### 4.4. Implications for Future Research

Bird plumage color change is a captivating field of avian research that has garnered attention for its significant roles. For our work, there are multiple dimensions of relevance in future research aimed at enhancing our understanding of plumage color changes of Crested Ibis. Foremost, there is a compelling need for an in-depth exploration of the molecular mechanisms underlying hormonal regulation. This includes investigating the specific molecular mechanisms of sex hormones, such as estrogens and testosterone, in the pathway of feather pigment synthesis [59]. This involves examining the specificity of sex hormone receptors, the interactions between hormones and related genes, as well as a detailed analysis of signal transduction pathways and regulatory elements. Secondly, an extensive investigation into pigment types [60,61,62] and synthesis pathways [63,64] is warranted. This primarily entails delving into different types of pigments, such as carotenoids, psittacofulvins, and melanins, and understanding their synthesis and transport mechanisms within bird species. Thirdly, it is imperative to study the influence of environmental factors on feather color changes [65,66,67]. This involves scrutinizing feather color variation in different ecosystems to comprehend how environmental factors impact avian feather coloration under varying seasonal and geographical conditions. Fourthly, an in-depth examination of the relationship between the plumage color change and visual signals is essential [68,69]. While our understanding of avian tetrachromatic vision is well established, further research is required to fully grasp how plumage coloration influences avian behavior and communication. Fifthly, the pivotal role of plumage color change in avian evolution necessitates comprehensive investigation [70,71]. Research into the evolutionary significance of plumage color changes provides insights into avian ecological adaptability and their evolutionary processes to survive in diverse environments.

Beyond the aforementioned research significance, we hold the following expectations regarding Crested Ibis’s plumage color change. The utilization of bioinformatics, genetic techniques, and transcriptome technologies can assist us in conducting a comprehensive analysis of the molecular mechanisms governing plumage color changes. By comprehending the genetic basis of coloration, we can achieve a deeper understanding of the mechanisms and influencing factors behind Crested Ibis plumage color changes.

## 5. Conclusions

In this study, our objective is to analyze the chemical composition of the black powdery substance and investigate its correlation with sex hormones. The results showed that the sex hormone levels regulate the secretion and application of the black substance. In females, the E2 level was highest during the courtship period, and the T level was highest during the brooding period, both of which were significantly correlated with daubing behavior (p=0.006<0.01). In males, the E2 level was generally low, but there was a significant correlation between the E2 level and daubing behavior (p=0.039<0.05), as well as a significant correlation between the T level and daubing behavior (p=0.003<0.01). In addition, we qualitatively analyzed the chemical components of the black substance secreted by crested ibis by gas chromatography–mass spectrometry (GC-MS). The results showed that the black substance contained a total of 117 kinds of chemical components, including esters, alkanes, alcohols, ketones, aldehydes, and other compounds.

## Figures and Tables

**Table 1:** Information on the seven captive-bred Crested Ibises, including their color plastic ring number (CPRN), gender, birth year, metal ring number (MRN), and feeding area.

	Color Plastic Ring Number	Gender	Birth -Year	Metal Ring Number	Feeding Area
For Hormone	543	♂	2006	7060	5-3
	491	♂	2005	7081	5-4
	461	♂	2004	7161	5-4
	443	♀	2004	7147	5-3
	334	♀	2003	2661	5-3
	603	♀	2007	2640	5-4
	527	♀	2005	5283	5-4
For Black Substance	307	♀	2002	NA	5-4
	501	♂	2005	7072	5-4

**Table 2:** Statistical data on daubing frequency, water bathing frequency, and the total daubing duration for Crested Ibis individuals (n=7) from January to July.

Month	January	February	March	April	May	June	July
Daubing Frequency	6	23	34	11	0	0	0
	4	25	34	33	2	0	0
	6	25	38	28	0	0	0
	6	20	36	32	0	0	0
	5	18	40	33	0	0	0
	3	20	38	35	0	0	0
	3	22	43	38	0	0	0
SD	1.38	2.67	3.26	8.91	0.76	0.00	0.00
Water Bathing Frequency	7	25	37	30	34	25	5
	5	28	36	35	38	28	8
	6	26	40	33	34	28	9
	7	24	38	35	32	24	8
	5	22	42	35	28	25	9
	7	23	40	38	26	22	6
	4	26	46	40	24	24	10
SD	1.21	2.04	3.39	3.24	5.01	2.19	1.77
The Total Duration of Daubing (Min)	42	188	313	78	0	0	0
	45	190	345	80	12	0	0
	46	205	330	76	0	0	0
	48	186	356	90	0	0	0
	43	198	346	85	0	0	0
	46	178	380	87	0	0	0
	36	179	400	96	0	0	0
SD	3.95	9.74	29.41	7.11	4.54	0.00	0.00

**Table 3:** Part results of constituents of the black substance by GC-MS analysis.

Categories	Compound Name and Molecular Formula	Compound Name and Molecular Formula
Ketones and aldehydes	Di-n-decylsulfone, $C_{20}H_{42}O_{2}S$	1-Oxaspiro[2.5]octan-4-one,2,2,6-trimethyl-, trans-, $C_{10}H_{16}O_{2}$
	4-Octanone, $C_{18}H_{16}O$	10-Nonadecanone, $C_{19}H_{38}O$
	5,10-Tetradecanedione, $C_{14}H_{26}O_{2}$	Cyclohexanone,3-(3,3-dimethylbutyl)-, $C_{12}H_{22}O$
	2-Nonenal, (E)-, $C_{9}H_{16}O$	2-Hexenal, 2-ethyl-, $C_{8}H_{14}O$
	(Z)-7-Hexadecenal,$C_{16}H_{30}O$	2(3H)-furanone,5-butyl-5-ethyldihydro- $C_{10}H_{18}O_{2}$
Esters	2-Aminopent-4-enoic acid, N-vinyloxycarbonyl-, nonyl ester, $C_{17}H_{29}NO_{4}$	Acetic acid, chloro-, isobutyl ester, $C_{6}H_{11}ClO_{2}$
	Decanoic acid, 2,3-dihydroxypropyl ester, $C_{13}H_{26}O_{4}$	3-Chlropropionic acid, nonyl ester, $C_{12}H_{23}ClO_{2}$
	Nonanoic acid, 6-phenyl-, methyl ester, $C_{16}H_{24}O_{2}$	Butanoic acid, 2-methyl-, octyl ester, $C_{13}H_{26}O_{2}$
	Succinic acid, 2-chloro-6-fluorophenyl non-3-en-1-yl ester, $C_{19}H_{24}ClFO_{4}$	1,3,5-Triazine-2,4(1H,3H)-dione,6-(ethylamino)-, $C_{5}H_{8}N_{4}O_{2}$
	Docosanoic acid, docosyl ester, $C_{44}H_{88}O_{2}$	9,12,15-Octadecatrienoic acid,2-phenyl-1,3-dioxan-5-yl ester, $C_{28}H_{40}O_{4}$
	Sulfurous acid, isohexyl 2-pentyl ester $C_{11}H_{24}O_{3}S$	Nonanoic acid, heptyl ester, $C_{16}H_{32}O_{2}$
	vinyl laurate, $C_{14}H_{26}O_{2}$	1,2-Benzenedicarboxylic acid, bis(2-methylpropyl) ester, $C_{16}H_{22}O_{4}$
Esters	cyclohexylmethyl hexadecyl ester, $C_{23}H_{46}O_{3}S$	Formic acid, 2,4,4-trimethylpentyl ester, $C_{9}H_{18}O_{2}$
	Eicosyl trifluoroacetate, $C_{22}H_{41}F_{3}O_{2}$	Heptacosyl acetate, $C_{29}H_{58}O_{2}$
	Sulfurous acid, octadecyl 2-propyl ester, $C_{24}H_{48}O_{3}S$	Diglycolic acid, 2-methylphenyl tridecyl ester, $C_{21}H_{44}O_{3}S$
	Bis(2-ethylhexyl) methylphosphonate, $C_{17}H_{37}O_{3}P$	Carbonic acid, decyl 2-ethylhexyl ester, $C_{19}H_{38}O_{3}$
	Eicosanoic acid, 15-oxo-, methyl ester, $C_{21}H_{40}O_{3}$	
Alcohols	2-Hexen-1-ol, (Z)-, $C_{6}H_{12}O$	trans-1,4-Cyclohexanediol, $C_{6}H_{12}O_{2}$
	1-Decanol, 2-hexyl-, $C_{16}H_{34}O$	E,E,Z-1,3,12-Nonadecatriene-5,14-diol, $C_{19}H_{34}O_{2}$
	Octacosanol, $C_{28}H_{58}O$	1,2-Cyclohexanediol,1-methyl-4-(1-methylethenyl)-, $C_{10}H_{18}O_{2}$
	1,2-Dihydrolinalool, $C_{10}H_{20}O$	
Hydrocarbons	Heptane, 3,3-dimethyl-, $C_{9}H_{20}$	Octane, 2-methyl-,$C_{9}H_{20}$
	Octane, 2,6-dimethyl-, $C_{10}H_{22}$	Tridecane, $C_{13}H_{28}$
	Undecane, 2, 6-dimethyl-, $C_{13}H_{28}$	Hexadecane, $C_{16}H_{34}$
	Nonane, 5-(2-methylpropyl)-, $C_{13}H_{28}$	2,6,10-Trimethyltridecane,$C_{16}H_{34}$
	Nonane, 3-methyl-5-propyl-, $C_{13}H_{28}$	Tridecane, 2-methyl-, $C_{14}H_{30}$
	Tetradecane, $C_{14}H_{30}$	
	3,5-Dimethyldodecane, $C_{14}H_{30}$	1H-Indene, octahydro-, cis-, $C_{9}H_{16}$
	Tetradecane, 4-methyl-, $C_{15}H_{32}$	Undecane, 3, 6-dimethyl-, $C_{13}H_{28}$
	Heneicosane, $C_{21}H_{44}$	Heptadecane, cis-, $C_{17}H_{36}$
	2, 6, 10, 15-tetramethyl-,$C_{16}H_{34}$	Tetradecane, 5-methyl-, $C_{15}H_{32}$
	Heptadecane, 2,6,10,15-tetramethyl-, $C_{21}H_{44}$	Decane, 5-ethyl-5-methyl-, $C_{13}H_{28}$
	Heptadecane, 8-methyl-, $C_{18}H_{38}$	Undecane, 6-cyclohexyl-, $C_{17}H_{34}$
	Undecane, 3-methyl-, $C_{12}H_{26}$	Nonadecane, $C_{19}H_{40}$
	Pentadecane, 2,6,10-trimethyl-, $C_{18}H_{38}$	Eicosane, $C_{20}H_{42}$
	Decane, 3,8-dimethyl-, $C_{12}H_{26}$	5-Butyl-5-ethylpentadecane, $C_{21}H_{44}$
	Pentadecane, 2,6,10,14-tetramethyl-, $C_{19}H_{40}$	2-methyloctacosane, $C_{29}H_{60}$
	Octadecane, $C_{18}H_{38}$	Hexadecane, 2,6,10,14-tetramethyl-, $C_{20}H_{42}$
	Tetracosane, $C_{24}H_{50}$	2-Methylhexacosane, $C_{27}H_{56}$
	Nonacosane, $C_{29}H_{60}$	Squalane, $C_{30}H_{62}$
	Hexatriacontane, $C_{36}H_{74}$	1-Cyclopentyleicosane, $C_{25}H_{50}$
	5-Butyl-5-ethylheptadecane, $C_{23}H_{48}$	Dodecylcyclohexane, $C_{18}H_{36}$
	2-Pentadecane, 2,6,10,14-tetramethyl-, $C_{19}H_{40}$	2-Methyltetracosane, $C_{25}H_{52}$
	Pentacosane, $C_{25}H_{52}$	15-Isobutyl-(13.alpha.H)-isocopalane, $C_{24}H_{44}$
	2,2,4,4,6,6,8,8-Heptamethyl-1-nonene, $C_{15}H_{30}$	7-Tetradecene, $C_{24}H_{44}$
	9-Octadecene, (E)-, $C_{18}H_{36}$	2,4-Dimethyl-1-hexene, $C_{8}H_{16}$
	Octadecane, 3-methyl-, $C_{19}H_{40}$	
Others	Butanoyl Chloride, 3-methyl-, $C_{5}H_{9}ClO$	Cyclotetrasiloxane, octamethyl-, $C_{8}H_{24}O_{4}Si_{4}$
	Anabasine, $C_{10}H_{14}O_{2}$	2-Propen-1-amine, N-2-propenyl-, $C_{6}H_{11}N$
	(1S, 2S)-(+)-1, 2-Diaminocyclohexane, $C_{6}H_{14}N_{2}$	Cycloheptasiloxane, tetradecamethyl-, $C_{14}H_{42}O_{7}Si_{7}$
	Azocine, octahydro-, $C_{7}H_{15}N$	(R)-2,4-Dihydroxy-N-(3-hydroxypropyl)-3,3-dimethylbutyramide, $C_{9}H_{19}NO_{4}$
Others	Disulfide, di-tert-dodecyl, $C_{24}H_{50}N_{2}$	Cyclohexasiloxane, dodecamethyl-, $C_{12}H_{36}O_{6}Si_{6}$
	Piperazine, 1,4-bis(5-methyl-1,2,3-2H-triazol-4-yl)-, $C_{10}H_{16}N_{8}$	1-Bromoeicosane, $C_{20}H_{41}Br$
	4-O-.beta.-D-galactopyranosyl-, $C_{12}H_{22}O_{11}$	Cycloheptasiloxane, tetradecamethyl-, $C_{14}H_{42}O_{7}Si_{7}$
	Phenol, 3,5-bis(1,1-dimethylethyl)-, $C_{14}H_{22}O$	Cyclooctasiloxane, hexadecamethyl,$C_{16}H_{48}O_{8}Si_{8}$
	Chlorguanide,$C_{11}H_{16}ClN_{5}$	Octadecane, 1-chloro-, $C_{18}H_{37}Cl$
	2-Bromotetradecane, $C_{14}H_{29}Br$	Hexacosyl nonyl ether, $C_{35}H_{72}O$
	Cyclodecasiloxane, eicosamethyl-, $C_{20}H_{60}O_{10}Si_{10}$	Octacosane, 1-iodo-, $C_{28}H_{57}I$
	Dotriacontane, 1-iodo-, $C_{32}H_{65}I$	Cyclononasiloxane, octadecamethyl-, $C_{18}H_{54}O_{9}Si_{9}$
	Triacontane, 1-bromo-, $C_{30}H_{61}Br$	Heptasiloxane, hexadecamethyl-, $C_{16}H_{48}O_{6}Si_{7}$
	2(3H)-Furanone, 4,5-dihydro-5-methoxy-4-(2,3-dimethyl-2-buten-4-yl)-, $C_{11}H_{18}O_{3}$	

## Data Availability

The study data were not deposited in an official repository. For access to the data supporting the study findings, please contact the authors directly.
